# Draft Genome Sequence of *Methylocapsa palsarum* NE2^T^, an Obligate Methanotroph from Subarctic Soil

**DOI:** 10.1128/genomeA.00504-17

**Published:** 2017-06-15

**Authors:** Kirill K. Miroshnikov, Alena Didriksen, Daniil G. Naumoff, Marcel Huntemann, Alicia Clum, Manoj Pillay, Krishnaveni Palaniappan, Neha Varghese, Natalia Mikhailova, Supratim Mukherjee, T. B. K. Reddy, Chris Daum, Nicole Shapiro, Natalia Ivanova, Nikos Kyrpides, Tanja Woyke, Svetlana N. Dedysh, Mette M. Svenning

**Affiliations:** a Winogradsky Institute of Microbiology, Research Center of Biotechnology RAS, Moscow, Russia; b UiT The Arctic University of Norway, Department of Arctic and Marine Biology, Tromsø, Norway; c Los Alamos National Laboratory, Joint Genome Institute, Biosciences Division Genome Science B6, Los Alamos, New Mexico, USA

## Abstract

*Methylocapsa palsarum* NE2^T^ is an aerobic, mildly acidophilic, obligate methanotroph. Similar to other *Methylocapsa* species, it possesses only a particulate methane monooxygenase and is capable of atmospheric nitrogen fixation. The genome sequence of this typical inhabitant of subarctic wetlands and soils also contains genes indicative of aerobic anoxygenic photosynthesis.

## GENOME ANNOUNCEMENT

*Methylocapsa palsarum* NE2^T^ is a methanotroph of the class *Alphaproteobacteria* family *Beijerinckiaceae*. It was isolated from a *Sphagnum*-covered collapsed palsa soil in northern Norway (1). Cells of *M. palsarum* are Gram-negative, nonmotile, nonpigmented, slightly curved thick rods that multiply by binary fission. The cells possess only a particulate methane monooxygenase (MMO); the growth substrates are methane and methanol. *M. palsarum* NE2^T^ is a mildly acidophilic, psychrotolerant methanotroph, which is capable of atmospheric nitrogen fixation under reduced oxygen tension.

The draft genome sequence was generated at the Department of Energy, Joint Genome Institute (JGI), using Illumina technology (2). An Illumina 300-bp insert standard shotgun library was constructed and sequenced using the Illumina HiSeq-2500 1 TB platform which generated 9,302,374 reads totaling 1,395.4 Mb. All general aspects of library construction and sequencing performed at the JGI can be found at http://www.jgi.doe.gov. All raw Illumina sequence data was filtered using BBDuk (http://sourceforge.net/projects/bbmap); artifact filtered Illumina reads were assembled using SPAdes (version 3.6.2) (3). Structural and functional annotations were performed using the JGI’s microbial genome annotation pipeline (4) and the genome was released through the Integrated Microbial Genomes (IMG) system (5). The final draft assembly contained 78 contigs. The total estimated size of the *M. palsarum* NE2^T^ genome is 4.105 Mb (coverage of 339.9×), with G+C content of 61.7%. A single rRNA operon, 47 tRNAs, and 3,820 protein-coding genes were identified.

The genome of *M. palsarum* NE2^T^ contains a single *pmoCAB* operon for particulate MMO; two additional *pmoC* homologues are also present. The absence of *mmo* operon encoding soluble MMO is typical for all *Methylocapsa* species (6, 7). Methanol oxidation capabilities are explained by the presence of two gene operons encoding MxaFI- and XoxF-methanol dehydrogenases (8). Genes involved in tetrahydromethanopterin-linked C1 transfer and formate oxidation were also identified. Similar to other *Beijerinckiaceae* methanotrophs (7), *M. palsarum* NE2^T^ possesses the complete set of genes for the function of the Calvin-Benson-Bassham cycle and the serine pathway for carbon assimilation, as well as genes encoding enzymes of the tricarboxylic acid cycle. The genes involved in N_2_ fixation are organized as in *Methylocapsa acidiphila* B2^T^ into one cluster (SAMN05444581_101312 to SAMN05444581_101348). Despite being described as a nonmotile bacterium, *M. palsarum* NE2^T^ possesses a number of genes required for the flagellum assembly. Surprisingly, this methanotroph also harbors genes indicative of aerobic anoxygenic photosynthesis. This array of genes in strain NE2^T^ is highly similar to that in many plant-associated *Methylobacterium* species (9) and includes genes encoding the light-harvesting complex (*pufABCML*), the reaction center (*puhA*), as well as genes involved in biosynthesis of bacteriochlorophyll and carotenoids. Most of these photosynthesis-related genes are assembled in gene clusters (SAMN05444581_1147 to SAMN05444581_1149, SAMN05444581_11410 to SAMN05444581_11450). It remains to be seen if these genes are expressed under some certain environmental conditions.

### Accession number(s).

The *Methylocapsa palsarum* NE2^T^ genome sequence was deposited in GenBank under the accession no. FOSN00000000. The version described in this paper is the first version, FOSN00000000.1.
